# Bioactivity of human adult stem cells and functional relevance of stem cell-derived extracellular matrix in chondrogenesis

**DOI:** 10.1186/s13287-023-03392-7

**Published:** 2023-06-14

**Authors:** Yangzi Jiang, Rocky S. Tuan

**Affiliations:** 1 Institute for Tissue Engineering and Regenerative Medicine, School of Biomedical Sciences, Faculty of Medicine, The Chinese University of Hong Kong, Shatin, Hong Kong SAR China; 2 Center for Neuromusculoskeletal Restorative Medicine, Hong Kong Science Park, Shatin, New Territories, Hong Kong SAR, China; 3 Center for Cellular and Molecular Engineering, Department of Orthopaedic Surgery, University of Pittsburgh School of Medicine, Pittsburgh, PA 15219 USA

**Keywords:** Adult stem cells, Adipose-derived stem cells, Bone marrow-derived stem cells, Cartilage-derived stem/progenitor cells, Extracellular matrix, Chondrogenesis, Cartilage repair

## Abstract

**Background:**

Autologous chondrocyte implantation (ACI) has been used to treat articular cartilage defects for over two decades. Adult stem cells have been proposed as a solution to inadequate donor cell numbers often encountered in ACI. Multipotent stem/progenitor cells isolated from adipose, bone marrow, and cartilage are the most promising cell therapy candidates. However, different essential growth factors are required to induce these tissue-specific stem cells to initiate chondrogenic differentiation and subsequent deposition of extracellular matrix (ECM) to form cartilage-like tissue. Upon transplantation into cartilage defects in vivo, the levels of growth factors in the host tissue are likely to be inadequate to support chondrogenesis of these cells in situ. The contribution of stem/progenitor cells to cartilage repair and the quality of ECM produced by the implanted cells required for cartilage repair remain largely unknown. Here, we evaluated the bioactivity and chondrogenic induction ability of the ECM produced by different adult stem cells.

**Methods:**

Adult stem/progenitor cells were isolated from human adipose (hADSCs), bone marrow (hBMSCs), and articular cartilage (hCDPCs) and cultured for 14 days in monolayer in mesenchymal stromal cell (MSC)–ECM induction medium to allow matrix deposition and cell sheet formation. The cell sheets were then decellularized, and the protein composition of the decellularized ECM (dECM) was analyzed by BCA assay, SDS-PAGE, and immunoblotting for fibronectin (FN), collagen types I (COL1) and III (COL3). The chondrogenic induction ability of the dECM was examined by seeding undifferentiated hBMSCs onto the respective freeze-dried solid dECM followed by culturing in serum-free medium for 7 days. The expression levels of chondrogenic genes *SOX9*, *COL2*, *AGN*, and *CD44* were analyzed by q-PCR.

**Results:**

hADSCs, hBMSCs, and hCDPCs generated different ECM protein profiles and exhibited significantly different chondrogenic effects. hADSCs produced 20–60% more proteins than hBMSCs and hCDPCs and showed a fibrillar-like ECM pattern (FN^high^, COL1^high^). hCDPCs produced more COL3 and deposited less FN and COL1 than the other cell types. The dECM derived from hBMSCs and hCDPCs induced spontaneous chondrogenic gene expression in hBMSCs.

**Conclusions:**

These findings provide new insights on application of adult stem cells and stem cell-derived ECM to enhance cartilage regeneration.

**Supplementary Information:**

The online version contains supplementary material available at 10.1186/s13287-023-03392-7.

## Introduction

Articular cartilage is not a self-repairable tissue; injuries to joint cartilage usually result in degenerative joint diseases (e.g., osteoarthritis) and mobility loss. Autologous chondrocyte-based therapies, such as autologous chondrocyte implantation, have been used successfully in clinical applications for more than two decades as an early regenerative intervention for articular cartilage defects [1]. However, the application of this cell-based therapy has been limited by donor tissue site morbidity and inadequate cell numbers in severe diseased joints due to the limited availability of healthy cartilage. Adult stem cells have therefore been proposed as a solution to the problem of inadequate donor cells seen in autologous chondrocyte-based therapies.

Multipotent stem/progenitor cells derived from autologous adipose tissue (human adipose-derived stem cells [hADSCs]), autologous bone marrow (human bone marrow-derived stem cells [hBMSCs]), and autologous articular cartilage (human cartilage-derived progenitor cells [hCDPCs]) [2, 3] are the most promising candidates for application in clinical cartilage repair due to their abilities of self-renewal and chondrogenic differentiation. However, the multi-lineage differentiation ability of certain stem cell types is not a guarantee for tissue regeneration ability. In practice, chondrogenic differentiation protocols are specially developed for each cell type and its derivatives. Sequential combinations of essential growth factors are required for the chondrogenic differentiation of hBMSCs and hADSCs and nascent cartilage maturation [4]. For instance, three successive chronological stages are involved in chondrogenesis and cartilage formation by hBMSCs: (1) cell condensation phase; (2) matrix deposition phase; and (3) tissue homeostasis phase (anti-hypertrophy and anti-endochondral ossification), with different groups of growth factors required at each stage. Specifically, fibroblast growth factors (FGFs) 2, 9, and 18 are essential for the cell condensation phase [5]; transforming growth factor β1 (TGFβ1) and growth differentiation factor 5 (GDF5) are required for cell condensation and matrix deposition, but long exposures to TGFβ1 can cause nascent cartilage hypertrophy and endochondral ossification [6]. Other reported chondrogenic growth factors include bone morphogenetic protein (BMP)2, BMP4, BMP7, and TGFβ3 [7–10], which are required by a variety of mesodermal cells [11, 12], and have multiple functions, such as osteogenic induction [13, 14]. In addition, hADSCs require more sophisticated combinations of growth factors for chondrogenic differentiation. For instance, BMP6 is needed together with other BMPs and/or TGFβs for successful hADSC chondrogenesis [15, 16]. To effectively use stem/progenitor cells isolated from articular cartilage and significantly enhance matrix deposition, the addition of BMP4 and TGFβ3 to the culture medium is required, although hCDPCs can also spontaneously form cartilage tissue in vivo [2, 17]. These stage- and cell type-specific growth factor repertoires likely arise due to the unique biological characteristics of stem cell types. As the host tissue usually produces inadequate amounts of the required growth factors, when these cells are implanted into the defect site for in situ cartilage repair and regeneration, the regenerative ability of these stem cells is difficult to predict. How engrafted stem/progenitor cells contribute to tissue regeneration and affect the quality of nascent cartilage formed from the engrafted cells, a process that is dependent on the remodeling of the cell-derived extracellular matrix (ECM), is not well understood.

The matrix of articular cartilage is rich and less cellular; the main components of articular cartilage ECM are type II collagen (COL2) and proteoglycans such as aggrecan (AGN). Cartilage tissue is formed during joint development from mesodermal stem cells, and the genes involved in chondrogenic differentiation and cartilage formation include *SOX9*, and *COL2*, *AGN*, and *CD44*. In the case of stem cell-based cartilage regeneration, adult stem cells could possibly contribute to cartilage repair by directly differentiation and integration, as well as paracrine effects [18], and ECM produced and deposited by transplanted stem cells may be one of the major contributors. The major components of MSCs derived ECM include fibronectin, type I collagen (COL1), and many others, depending on the cell type and cell culture conditions [19]. To further analyze the ECM deposited by different types of human adult stem cells, and their effect on cartilage tissue formation, in this study, we evaluated the bioactivity, particularly the chondrogenic induction ability, of the ECM produced by adult tissue-specific stem/progenitor cells derived from human adipose, bone marrow, and articular cartilage tissue and report the found of significant differences across the cell types. Such information could provide new insights that can help improve the quality of regenerated tissues and enhance the applicability of adult stem cell-based therapies for cartilage repair.

## Materials and methods

### Adult stem cell isolation, culture, and characterization

Adult human adult stem/progenitor cells were isolated from adipose (hADSC, lipoaspirate, biological donors *n* = 3, age 34–43 years, from both sexes), bone marrow (hBMSC, hip arthroplasty, biological donors *n* = 6, age 53–59 years, from both sexes), and articular cartilage tissue (hCDPC, knee arthroplasty, biological donors *n* = 4, age 47–66 years, from both sexes) with approval from the Institutional Review Board (University of Pittsburgh and University of Washington as published in [2]; The Chinese University of Hong Kong). The clone-forming ability, multipotency, and stem cell-related surface markers have been characterized and partially documented in our previously publications (hADSCs [20], hBMSCs [2, 21], and hCDPCs [2]). To reduce donor-to-donor variation, the same number of cells from multiple donors was pooled at Passage 1 (P1) and expanded to P2 in growth medium (Dulbecco’s modified Eagle medium–high glucose; 10% fetal bovine serum [FBS]; 100 units/mL penicillin and 100 mg/mL streptomycin; all of the reagents used in the current study were obtained from Thermo Fisher Scientific [Massachusetts, USA] unless otherwise stated) and stored in liquid nitrogen for further study.

### Cell sheet formation, ECM preparation and decellularization

Pooled hADSCs (*n* = 3 per batch), hBMSCs (*n* = 3 per batch), and hCDPCs (*n* = 4 per batch) were seeded into 6-well plates (1 × 10^5^ cells per well) and cultured in mesenchymal stromal cell (MSC)–ECM induction medium (growth medium with 50 μM ascorbic acid, as described in our earlier publication [19]) for 14 days to allow matrix deposition and cell sheet formation. The cell sheets were then collected to serve as cell sheet controls for the decellularized groups.

To remove the bioactivity of cellular components, the cell sheets formed by hADSCs, hBMSCs, and hCDPCs were decellularized by treatment with a 0.5% Triton X-100 (Sigma) solution containing 20 mM NH_4_OH in phosphate-buffered saline (PBS) for 5 min. They were then washed with PBS and treated with 100 unit/mL DNase and RNase (Worthington, Columbus, Ohio, USA) at room temperature for 1 h to remove DNA and RNA. The clearing of DNA from the decellularized ECM (dECM) was verified by staining cell nuclei with 4′,6-diamidino-2-phenylindole (DAPI) and using the Picogreen assay (data not shown in figures; the clearance of cellular components can be supported by the results in Fig. 4—intracellular components were undetectable in the dECM groups). The decellularized cell sheets were then either stored in liquid nitrogen or freeze-dried using a lyophilizer and stored at 4 °C until further usage.

### Protein concentration and sodium dodecyl sulfate–polyacrylamide gel electrophoresis (SDS-PAGE) analysis

Proteins from the cell sheet control and dECM groups were extracted using 200 μL/well radioimmunoprecipitation assay (RIPA) buffer (Sigma) and a protease inhibitor cocktail (Sigma). The protein content of the cell sheets (generated by seeding 1 × 10^5^ cells per well) and dECM was determined by the bicinchoninic acid (BCA) assay (Pierce BCA protein assay kit, Thermo Fisher). The total protein components were analyzed by SDS-PAGE (5 μg protein per lane of both the cell sheet and dECM samples) with Coomassie blue staining [22]. Briefly, equal amounts of protein from each group were mixed with SDS loading buffer, either treated or not treated with reducing agent (NuPAGE; Life Technologies, Carlsbad, CA, USA), and heated for 10 min at 70 °C. The protein was loaded onto a pre-cast 10-well NuPAGE 4–12% Bis–Tris Minigel (Life Technologies) and separated by electrophoresis in 3-(*N*-morpholino)propane sulfonic acid running buffer for 50 min at a constant voltage of 200 V. The gel was stained with SimplyBlue™ SafeStain (Life Technologies) for 2–4 h and then washed in water multiple times until the background became clear. It was photographed using a charge-coupled device (CCD) camera gel imaging system (Fotodyne, Hartland, WI, USA).

### Chondrogenic induction assay by quantitative mRNA expression

The stem cell chondrogenic induction ability of the dECM generated by hADSCs, hBMSCs, and hCDPCs was evaluated by seeding 1 × 10^5^ hBMSCs (*n* = 3, pooled from three donors distinct from the ECM donor) onto freeze-dried, solid dECM. Briefly, 1 × 10^5^ cells in 50 μL serum-free growth medium (DMEM-high glucose; insulin–transferrin–selenium [ITS]; 100 units/mL penicillin and 100 mg/mL streptomycin) were seeded onto dry dECM in a 1.5-mL tube and incubated at 37 °C and 5% CO_2_ in a cell culture incubator for 30 min; 1 mL serum-free growth medium was then added carefully, and the tubes were incubated for a further 8 h in cell culture incubator at 37 °C and 5% CO_2_ to allow cell attachment. To reduce the effect of unattached self-assembled hBMSC pellets on gene expression, the dECM constructs with attached hBMSCs were transferred to new tubes and cultured in serum-free growth medium (DMEM-high glucose; ITS; 100 units/mL penicillin and 100 mg/mL streptomycin) in cell culture incubator at 37 °C and 5% CO_2_ for 7 more days, and the serum-free growth medium was changed once at day 3. The cell-dECM cultures were then transferred to collecting tubes for further analysis. The expression of chondrogenic/cartilage matrix-related genes (*SOX9*, *COL2*, *AGN*, and *CD44*) was analyzed by quantitative polymerase chain reaction (q-PCR). *18SrRNA* and *RPL13a* were used as housekeeping genes and monolayer-cultured hBMSCs in 6-well plate were used as controls in this assay.

### RNA extraction and quantitative real-time (RT)-PCR

RNA was isolated using the RNeasy Mini Kit (Qiagen) in accordance with the manufacturer’s instructions. Quantitative RT-PCR was performed using the SYBR Green PCR Master Mix (Life Technologies) and a StepOne Plus Realtime PCR system (Applied Biosystems). The PCR protocol consisted of 45 cycles of template DNA amplification with primer annealing conducted at 60 °C. Relative gene expression was calculated using the 2-delta delta Ct method. *18SrRNA* and *RPL13a* were used as housekeeping genes for the monolayer and cell-attached dECM, both of which yielded similar results. The primer sequences used in this study are listed in the Additional file 1: Table S1.

### Immunoblotting

Proteins were extracted using RIPA buffer (Sigma) and a protease inhibitor cocktail (Sigma), boiled in sample buffer (Bio-Rad), and separated by SDS-PAGE using a 6% stacking gel and 4–12% separating gel (5 μg protein/lane) under reducing conditions, i.e., the disulfide bonds were removed (NuPAGE; Life Technologies). Polyvinylidene fluoride (0.45 μm; Millipore) blots were prepared and incubated overnight at 4 °C with primary antibodies (collagen type I [COL1, ab34710] and collagen type III [COL3, ab184993], fibronectin [FN, ab281575]; 1:1000; Abcam), followed by enzyme-conjugated secondary antibodies (GE). The signal was detected using an ECL Kit (Pierce) and visualized using a FOTO/Analyst1 Fx CCD imaging system (Fotodyne). The images were analyzed using Image J (NIH). Each blot was repeated at least twice, and representative scans are presented.

### Statistical analysis

Each experiment was performed independently at least three times, with at least three technical replicates. The data are presented as mean ± standard deviation (SD) from representative trials. A one-way or two-way analysis of variance (ANOVA), followed by the least significant difference (LSD) post hoc test, was used to assess the statistical significance of multiple-group comparisons in GraphPad Prism (v7, GraphPad Software, San Diego, CA). Results with *p* < 0.05 were considered statistically significant.

## Results

### ECM protein production and deposition in adipose, bone marrow, and cartilage tissue-derived stem/progenitor cells

**Fig. 1:**
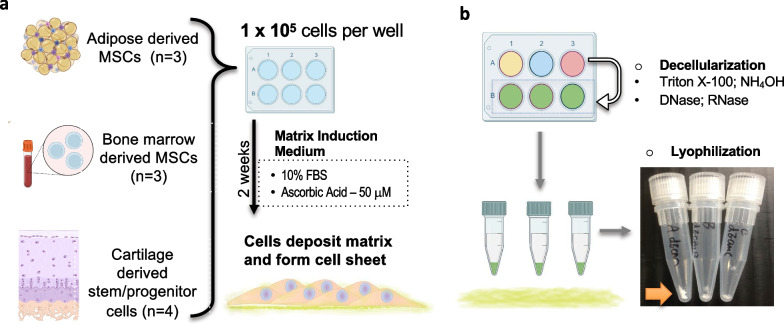
** Schematic diagram of stem cell-derived cell sheet and dECM sample preparation**. **a**. Adult human adult stem/progenitor cells were isolated from adipose (hADSC, lipoaspirate, donors *n* = 3), bone marrow (hBMSC, hip joint replacement, donors *n* = 3), and articular cartilage tissue (hCDPC, knee joint replacement, donors *n* = 4) with Institutional Review Board approval. The cells were pooled and expanded to Passage 2. hADSCs, hBMSCs, and hCDPCs were seeded into 6-well plates (1 × 10^5^ cells/well) and cultured in regular MSC–ECM induction medium (growth medium with 50 µM ascorbic acid) for 14 days to allow matrix deposition and cell sheet formation. **b**. The cell sheets were decellularized by treatment with Triton X-100, NH_4_OH, DNase, and RNase. The decellularized cell sheets were then freeze-dried using a lyophilizer and stored at 4 °C until further usage. The bottom right shows the morphology of freeze-dried decellularized cell sheets in 1.5-mL tubes for each group, and the arrow indicates the dECM derived from 1 × 10^5^ hADSCs, hBMSCs, and hCDPCs (left to right). *hADSCs*, human adipose-derived stem cells; *hBMSCs*, human bone marrow-derived stem cells; *hCDPCs*, human cartilage-derived progenitor cells; *ECM*, extracellular matrix; *dECM*, decellularized ECM; *MSC*, mesenchymal stromal cell. The schematic diagrams were created with BioRender.com by the authors with license obtained

**Fig. 2:**
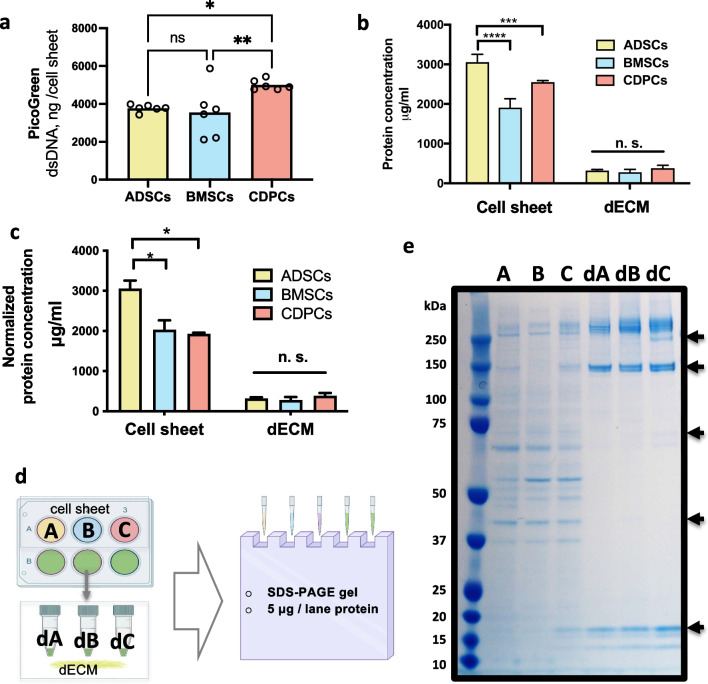
**ECM proteins produced by hADSCs, hBMSCs, and hCDPCs**. The protein content and components of the cell sheets and dECM were determined by the BCA assay and SDS-PAGE. **a**. PicoGreen readouts of double-stranded DNA (dsDNA) amount of the cell sheets generated from different stem cells. **b**. hADSCs produced significantly higher amounts of protein than hBMSCs and hCDPCs. Group A: 3054.3 ± 196.3 μg/mL; group B: 1909.3 ± 221.9 μg/mL; group C: 2552.7 ± 37.9 μg/mL; ****p < 0.0001; ***p = 0.0005. Group dA, 321.0 ± 27.8 μg/mL; group dB, 278.5 ± 71.7 μg/mL; group dC, 381.8 ± 74.3 μg/mL; not significant between the dECM groups, *p* > 0.3. The experiment was repeated three times, with at least three technical replicates for each batch. One representative batch is shown, and the data are presented as mean ± SD. A two-way ANOVA, followed by LSD, was used for statistical analysis, and *p* < 0.05 was considered statistically significant. **c.** The BCA protein amount readouts of cell sheet and dECM groups, the cell sheet groups were normalized to PicoGreen dsDNA readouts, *p < *0.01*; **d**. Diagram of the loading strategy used for SDS-PAGE. The samples were normalized to the protein concentration, and 5 μg protein was loaded into each lane of the gel. **e**. The SDS-PAGE results indicate the different ECM protein profiles (specifically at the ~ 10–20, 60, 75, 140, 230, and 260 kDa zones) of hADSCs, hBMSCs, and hCDPCs. The experiment was repeated at least three times, with at least two technical replicates for each trial. A representative trial is presented. A, cell sheet formed by hADSCs; *dA*, dECM from hADSC cell sheet; *B*, cell sheet formed by hBMSCs; *dsDNA*, double-stranded DNA; *dB*, dECM from hBMSC cell sheet; *C*, cell sheet formed by hCDPCs; *dC*, dECM from hCDPC cell sheet. *hADSCs*, human adipose-derived stem cells; *hBMSCs*, human bone marrow-derived stem cells; *hCDPCs*, human cartilage-derived progenitor cells; *ECM*, extracellular matrix; *dECM*, decellularized ECM; *BCA*, bicinchoninic acid, *SD*, standard deviation; *ANOVA*, analysis of variance; *LSD*, least significant difference; *SDS-PAGE*, sodium dodecyl sulfate–polyacrylamide gel electrophoresis. Figure 2d was created with BioRender.com by the authors with license obtained

To compare ECM protein production and deposition by different stem cell types, we used basal medium with ascorbic acid to enrich matrix production by human adult stem/progenitor cells that were derived from adipose (hADSC, *n* = 3, group A), bone marrow (hBMSC, *n* = 3, group B), and articular cartilage tissue (hCDPC, *n* = 4, group C). We cultured 1 × 10^5^ cells in 6-well plates for 14 days to obtain a matrix-rich cell sheet (Fig. 1a) and obtained around 2–4 × 10^5^ cells per well after they formed cell sheets (Fig. 2a). Stem cell-generated ECM was collected from the decellularized and lyophilized cell sheets, as shown in Fig. 1b.

### Protein quantification and SDS-PAGE analysis

To quantify the protein produced by stem cells, cell sheets, and dECM, respective protein samples from hADSC, hBMSC, and hCDPCs-derived cell sheets were collected and evaluated using the BCA assay (Fig. 2b) and normalized to the dsDNA Picogreen readouts in the cell sheet groups (Fig. 2c). The hADSC cell sheets had significantly higher amounts of protein than the hBMSC or hCDPC cell sheets (Fig. 2c, group A: 3054.3 ± 196.3 μg/mL; group B: 2027.6 ± 235.6 μg/mL; group C: 1925.6 ± 28.6 μg/mL). As the decellularization steps removed a significant amount of protein from the cell sheets, there were no significant differences in the protein concentrations of the dECM groups (Fig. 2b, c; group dA, 321.0 ± 27.8 μg/mL; group dB, 278.5 ± 71.7 μg/mL; group dC, 381.8 ± 74.3 μg/mL).

We next analyzed the spectrum of protein expression in the cell sheet and dECM groups by SDS-PAGE (5 μg protein/lane) (Fig. 2d). The cell sheet groups showed a broad spectrum of proteins of multiple molecular weights, consistent of their derivation from both cellular and ECM proteins, with a slight differences between the groups (e.g., bands at 40–70 kDa and 140 kDa; Fig. 2e). In comparison, the dECM groups exhibited more intense protein bands at 140 and 260 kDa and also at a low molecular weight range between 10 and 20 kDa, but not in intermediate molecular weight range, consistent with removal of cellular proteins. Intense staining was seen at 140 and 260 kDa in all three dECM groups, with the dC group showing an additional unique band at 250 kDa (Fig. 2e). As these bands corresponded in molecular weight to COL and FN (140 and 260 kDa, respectively), immunoblotting for these two proteins was then carried out in later session.

### Chondrogenic induction by the dECM derived from hADSCs, hBMSCs, and hCDPCs

**Fig. 3:**
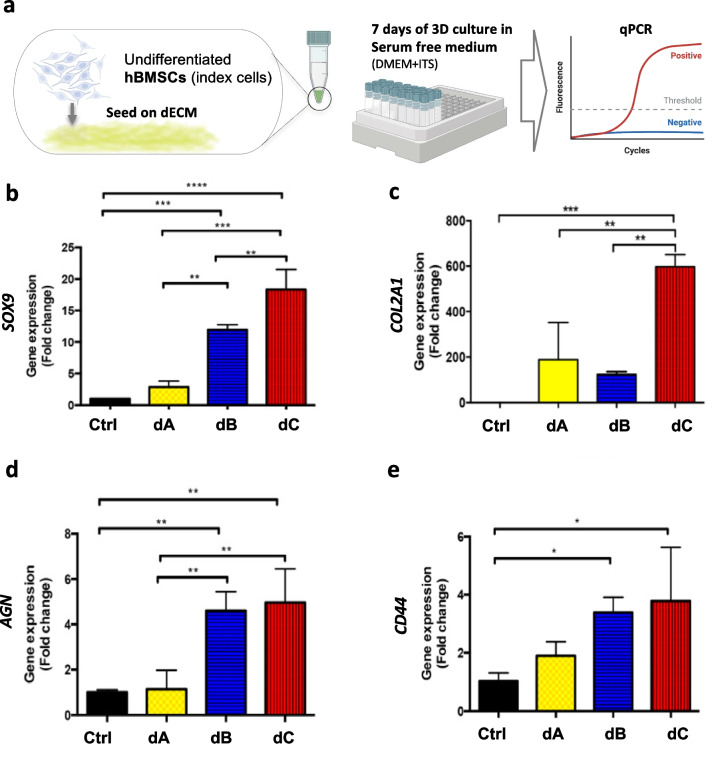
**Chondrogenic induction ability of the dECM generated by hADSCs, hBMSCs, and hCDPCs**. **a**. Experimental set up. To examine the chondrogenic induction ability of the dECM, undifferentiated hBMSCs were used as index cells and seeded atop the dECM derived from different cell sources. 1 × 10^5^ hBMSCs (*n* = 3, pooled from three donors other than the matrix donor) grown in serum-free medium were seeded onto freeze-dried dECM. The attached BMSC–dECM constructs were then moved to new tubes and cultured in serum-free growth medium (DMEM-high glucose and ITS) without the addition of any other growth factors for 7 days, followed by q-PCR examination. **b**–**e**. The expression levels of chondrogenic/cartilage matrix-related genes (*SOX9*, *COL2A1, AGN, CD44*, *HKG:18SrRNA*, and *RPL13a*; Control: monolayer-cultured BMSCs). The experiment was repeated at least three times, with three technical replicates (cell–dECM constructs) for each trial. The mRNA extracted from each cell–dECM construct was examined with three technical replicates in the q-PCR assay. Data are presented as mean fold changes ± SD from a representative trial. A one-way ANOVA, followed by LSD, was used for statistical analysis, and *p* < 0.05 was considered statistically significant. *Ctrl*, monolayer cultures of index BMSCs; *dA*, index BMSCs seeded onto the hADSC–dECM; *dB*, index BMSCs seeded onto the hBMSC–dECM; *dC*, index BMSCs seeded onto the hCDPC–dECM. *SOX9*, SRY-box transcription factor 9; *COL2A1*, pro-alpha1(II) chain of type II collagen; *AGN*, aggrecan; *CD44*, cell surface glycoprotein, receptor of hyaluronic acid; *18SrRNA*, 18S ribosomal RNA; *RPL13a*, ribosomal protein L13a. *hADSCs*, human adipose-derived stem cells; *hBMSCs*, human bone marrow-derived stem cells; *hCDPCs*, human cartilage-derived progenitor cells; *ECM*, extracellular matrix; *dECM*, decellularized ECM; *q-PCR*, quantitative polymerase chain reaction; *DMEM*, Dulbecco’s modified Eagle medium; *ITS*, insulin–transferrin–selenium; *SD*, standard deviation; *ANOVA*, analysis of variance; *LSD*, least significant difference. Figure 3a was created with BioRender.com by the authors with license obtained

To examine the chondrogenic induction ability of the dECM derived from hADSCs, hBMSCs, and hCDPCs, a group of undifferentiated hBMSCs (*n* = 3, pooled from three donors distinct from the ECM donor) were selected as differentiation indicator cells due to their multi-lineage differentiation ability. We seeded 1 × 10^5^ hBMSCs individually onto the dECM derived from hADSCs, hBMSCs, and hCDPCs (Fig. 3a). The cells were allowed to attach to the dECM for 30 min, and the hBMSC-dECM constructs were then moved to new tubes and cultured in serum-free growth medium without the addition of any chondrogenic growth factors for 7 days. Expression of the chondrogenic/cartilage matrix-related genes *SOX9*, *COL2*, *AGN*, and *CD44* was analyzed using q-PCR (Fig. 3b–e). Significantly upregulated expression of the chondrogenic differentiation transcription factor *SOX9*, cartilage matrix gene aggrecan *AGN*, and hyaluronic acid receptor *CD44* was seen in the hBMSC- and hCDPC-derived dECM cultures, compared to monolayer-cultured cells (*p* < 0.05). Expression of the cartilage matrix COL2 gene, *COL2*, was significantly higher in the hCDPC-derived dECM group than in other cell type-derived dECM groups (Fig. 3). These results suggested that the dECM derived from hBMSCs and hCDPCs, but not from hADSCs, represented a chondroinductive microenvironment that resulted in to chondrogenesis and cartilaginous matrix deposition of the newly seeded hBMSCs.

### ECM protein content and component analysis

**Fig. 4:**
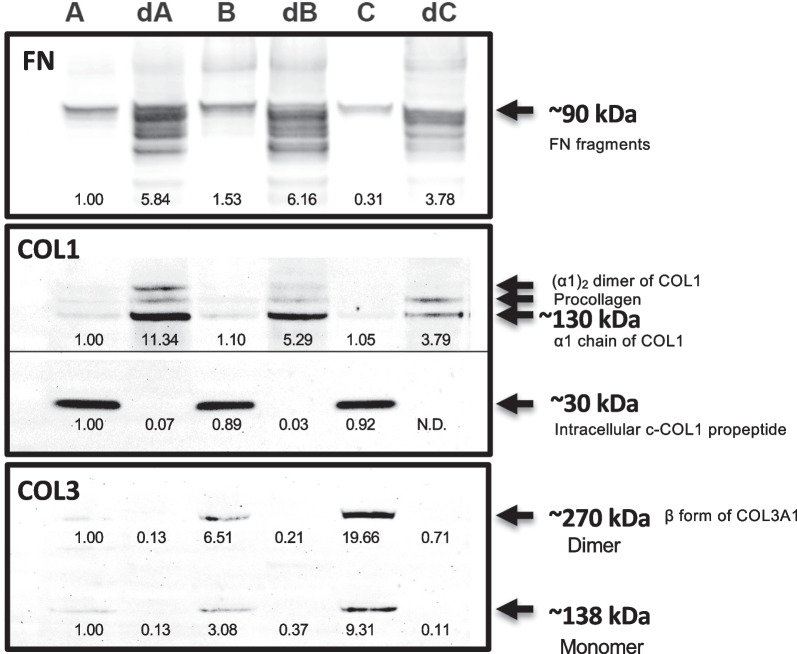
**Fibrillary components of FN, COL1, and COL3 in the stem cell-generated ECM**. Western blot results of FN, COL1, and COL3 in the cell sheets and dECM generated by hADSCs, hBMSCs, and hCDPCs. *A*, cell sheet formed by hADSCs; *dA*, dECM from hADSC cell sheet; *B*, cell sheet formed by hBMSCs; *dB*, dECM from hBMSC cell sheet; *C*, cell sheet formed by hCDPCs; *dC*, dECM from CDPC cell sheet. *FN*: fibronectin fragments, ~ 90 kDa (bands at ~ 263 kDa under non-reducing conditions can be found in Additional file 1: Fig. S1); *COL1*, collagen type I, intracellular c-terminal-COL1 propeptide can be found at ~ 30 kDa; mature ECM COL1 at ~ 130 kDa (arrows from bottom to top indicate the α1 chain of COL1, procollagen, and (α1)2 dimer of COL1, respectively); *COL3,* collagen type III, monomer at ~ 138 kDa, and dimer at ~ 270 kDa. The same amounts of protein (5 μg protein/lane) were loaded into each well, and SDS-PAGE was performed under reducing conditions. The experiment was repeated at least three times, with at least two technical replicates for each trial. The data were quantified using Image J (NIH, USA), normalized with group *A*, and a representative trial is presented. Full-length blots/gels are presented in Additional file 1: Figs. S2–S4. *hADSCs*, human adipose-derived stem cells; *hBMSCs*, human bone marrow-derived stem cells; *hCDPCs*, human cartilage-derived progenitor cells; *ECM*, extracellular matrix; *dECM*, decellularized ECM; *SDS-PAGE*, sodium dodecyl sulfate polyacrylamide gel electrophoresis

To further analyze the biochemical nature of the components responsible for the chondrogenic effects of the dECMs derived from different adult stem cells, SDS-PAGE followed by immunoblotting was carried out to detect the ECM proteins (FN, COL1, and COL3) (Fig. 4).

We loaded the same amount of proten to each lane, and among the cell sheet groups, more FN was detected in hBMSCs and hADSCs than in hCDPCs. In the dECM groups, the concentration of FN was higher in the dB and dA groups than in the dC group, with the two former groups also showing the presence of a number of immunoreactive FN fragments (details in Additional file 1: Fig. S2).

COL1 was detected in cell sheets formed by all three cell types, in the form of both proCOL1 and the mature protein. In the dECM, higher accumulation of mature COL1 (~ 130 kDa) was seen in the dB and dA groups than in the dC group (Note: procollagen and *β*-dimeric forms were also detected, Fig. 4; details in Additional file 1: Fig. S3). In addition, cleaved C-propeptide (35 kDa band) was clearly observed in all three cell sheet groups but not in the dECM groups, suggest a clearance of cellular parts in the dECM groups.

For COL3, among hADSCs, hBMSCs and hCDPCs, hCDPCs were found to produce significantly more COL3, and both monomeric and dimeric forms were detected at 138, and 270 kDa, respectively (Fig. 4; details in Additional file 1: Fig. S4). Although higher expression of COL3 was detected in hCDPC-derived cell sheets, the protein was almost undetectable in the dECM groups, suggesting that COL3 produced by the cells was likely mainly cell associated and not integrally associated with the ECM, and thus removed by decellularization.

Taken together, we found that the adult stem/progenitor cells derived from adipose tissue and bone marrow produce and deposit more FN and COL1 than cells derived from cartilage tissue.

## Discussion

In this study, we characterized the bioactivity, particularly the chondrogenic induction ability, of the ECM produced by adult stem/progenitor cells derived from human adipose, bone marrow, and articular cartilage tissue. Stem cells were cultured in MSC–ECM induction medium to form cell sheets. The ECM generated by hADSCs, hBMSCs, and hCDPCs was collected and then decellularized using an established protocol that retained the bioactivity of the stem cell-derived ECM [19] (Fig. 1). Different cells sources were observed to show different patterns of ECM deposition. hADSCs produced higher amounts of matrix proteins under the same cell culture conditions used for the other cell types (Fig. 2), but the chondrogenic induction ability of the hADSC-derived ECM was not as robust as that of the hBMSC- or hCDPC-derived ECM. The dECM derived from hBMSCs and hCDPCs showed spontaneous chondrogenic induction effects on undifferentiated hBMSCs. This property should be taken into account in cell source consideration in the application of adult stem cells for cartilage repair in vivo*.*

We next used undifferentiated hBMSCs as indicator cells to evaluate the chondrogenic induction ability of the dECM and monitored cell behaviors and profiled stage-specific markers known to represent the sequential events that occur during hBMSC chondrogenic differentiation [23]. The experiment was performed under three-dimensional, serum-free culture conditions (with ITS) without the addition of other known chondrogenic induction growth factors (e.g., TGFs and BMPs). Only the attached cells were cultured and examined, thus minimizing the influence of factors other than the chondrogenic induction effect of the dECM. Chondrogenic differentiation is characterized by the early expression of specific transcription factors, e.g., *SOX9,* followed by cartilage-specific ECM genes, e.g., *AGN*, and *COL2A1.* Interestingly, as shown in Fig. 3, the chondrogenic transcription factor *SOX9,* cartilage ECM gene *AGN*, and hyaluronic acid receptor *CD44* were all upregulated in the dB and dC groups, suggesting that the dECM derived from hBMSCs and hCDPCs could guide the attached hBMSCs in differentiating along the chondrogenic lineage. Specifically, the hCDPC-derived dECM enhanced the expression of the cartilage matrix gene *COL2* in the index hBMSCs, suggesting that the hBMSCs in the dC group not only initiated chondrogenic differentiation, but also entered the matrix-producing stage at day 7 in the in vitro cultures, considerably faster than in the other two groups (Fig. 3c).

To gain insight into potential identify of the bioactive components, we characterized the composition of the ECMs derived from the difference cell sources. As shown in Fig. 4 (Western blotting under reducing conditions) and Additional file 1: Fig. S1 (immunoblots performed under non-reducing conditions), significantly higher amounts of FN were found in the hBMSC- and hADSC-derived groups than in the hCDPC-derived groups, both in the cell sheets and dECM, suggesting a stronger pro-fibrillogenesis phenotype in the hADSCs and hBMSCs than in the hCDPCs. We also examined two types of collagen, COL1 and COL3, due to their contributions to ECM fibrillogenesis [24]. Multiple bands were observed in the collagen western blots under reducing conditions that disrupt the disulfide bonds responsible for trimeric formation during collagen biosynthesis and structural processing [25]. Disulfide bonds affect both the bioactivity and protein folding of collagen; namely, disulfide bonds connect the C-terminal pro-peptide of procollagen chains during intracellular assembly and triple helix formation of collagens [25–28]. All of the cells actively expressed COL1, and no significant differences in intracellular procollagen processing were found at the intracellular level, as indicated by the level of the C-propeptide (~ 30 kDa) in the cell sheets. However, we found that higher levels of mature COL1 were accumulated and deposited in the hADSC- and hBMSC-derived dECM (dA and dB) than in the hCDPCs-derived dECM (at ~ 130 kDa). On the other hand, we found higher expression levels of COL3 in hCDPCs than in hBMSCs and hADSCs, but COL3 did not acculmulate in the ECM (almost undetectable in the dECM groups, Fig. 4). It should be noted that the composition of the dA and dB ECM, namely presence of high levels of FN and COLI and low level of COL3, is similar to that reported in the early condensation phase of chondrogenesis in the embryonic limb bud [29]. Whether and how this ECM composition contributes to the pro-chondrogenesis activity of decellularized ECM of hADSC and hBMSC remains to be investigated.

**Fig. 5:**
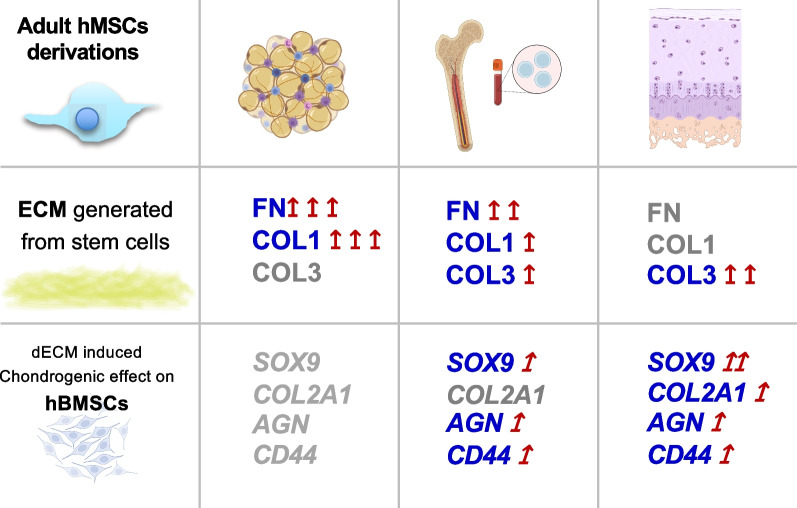
**Schematic of the study**. Schematic of the study. In this study, we characterized the bioactivity, particularly the chondrogenic induction ability, of the ECM produced by adult stem/progenitor cells derived from human adipose, bone marrow, and articular cartilage tissue. Our results suggest the presence of different in vivo cartilage regenerative potencies and patterns of adult stem cells derived from adipose, bone marrow, and cartilage tissue. *FN*: fibronectin; *COL1*, collagen type I; *COL3,* collagen type III; *SOX9*, SRY-box transcription factor 9; *COL2A1*, pro-alpha1(II) chain of type II collagen; *AGN*, aggrecan; *CD44*, cell surface glycoprotein, receptor of hyaluronic acid; *ECM*; extracellular matrix. Figure 5 was created with BioRender.com by the authors with license obtained

Taken together, our results indicate that the ECMs of adult stem cells derived from adipose, bone marrow, and cartilage tissue possess different chondrogenesis-promoting activities in vitro, suggesting potential different cartilage regenerative potencies in vivo (Fig. 5). Our findings suggest that: (1) hCDPC ECM, which has low COL1 and FN contents (Fig. 4), has the highest cartilage-promoting potency, indicated by the stimulation of high gene expression of two important cartilage matrix components, *ACN* and *COL2A1*, in the indicator hBMSC cultures (Fig. 3); (2) hBMSC ECM also enhances chondrogenic differentiation (Fig. 3) and aggrecan deposition, but the repaired tissue could potentially become fibrous due to the high abundance of FN (Fig. 4); and (3) the application of hADSCs may lead to rapid tissue filling and higher protein production by stem cells (Fig. 2c), but cartilage-like tissue formation is less likely due to the low chondrogenesis-promoting activity the hADSC ECM (Figs. 3, 4). These possible outcomes are in fact consistent with the fact that the implantation of hBMSCs [30–32] and hCDPCs [2] has been shown to achieve positive cartilage regeneration in animals [33] and in clinical trials to treat cartilage defect repair [30–32]. Most clinical reports of using hADSCs, in the form of adipose-derived stromal vascular fraction, together with biologics (e.g., fibrin and/or platelet-rich plasma) have been in the context of osteoarthritis treatment; these studies have shown some positive effects such as the reduction of joint pain and improvements in joint function [34]. However, the use of hADSCs alone may not be sufficient to achieve cartilage tissue regeneration [35].

There are, of course, limitations to this study. First, our minimization of the effect of in vitro culture systems on chondrogenic effects was not absolute. While we excluded exogenous chondrogenic factors in the cell culture medium to mimic natural in vivo condition, the presence of growth factors with chondrogenic effects, such as TGFβ1 in FBS [36], and insulin-like growth factor, i.e., ITS, in the serum-free culture medium to support basic cell survival and function must be noted. By using identical culture conditions (Fig. 1) and proper controls (Fig. 3), part of these concerns may be addressed, but potential autocrine effects of different stem cell types in long-term cultures [37] and the ability of the ECM to serve as a reservoir for these stem cell-derived growth factors [38, 39] remain to be answered. Next, although the cells used here displayed positive mesenchymal stem cell markers and multi-lineage differentiation capability [2, 20, 21], the limited biological donor number and the age range of the stem cell donors is also noted. We have attempted to address this limitation by using cells from three biological donors were used to represent each derivation of stem cells, as well as pooling the cells into batches to reduce individual differences. The slight age differences among the groups (A: 38–43 years; B: 53–59 years; C: 47–66 years) could also have affected the results. Therefore, larger sample groups with pairwise donor(s) are needed to further verify the findings reported here to further test the proposed hypothesis.

## Conclusion

In this study, we evaluated the bioactivity, particularly the chondrogenesis induction ability, of the ECM generated by hADSCs, hBMSCs, and hCDPCs. Although hADSCs produced high amounts of ECM proteins, the chondrogenic induction ability of the hADSC-derived ECM was significantly less than that of the hBMSC- or hCDPC-derived ECM. Our in vitro findings suggest potential differences in the cartilage repair capabilities of these adult stem/progenitor cells in applications in vivo. hCDPCs might form the best cartilage-like tissue, whereas hBMSCs can provide a chondrogenic induction environment with aggrecan and other matrix protein deposition in the repaired tissue. The application of hADSCs may lead to the quick formation of fibrous fillings, but with a lower probability of forming cartilage-like tissue. These findings should inform the selection of cell source and design of cell-based therapies for long-term cartilage repair.

## Supplementary Information

**Additional file 1**. **Supplementary Materials and Result. Table S1:** Primer sequences for real-time RT-PCR. **Fig. S1:** Western blot results of Fibronectin under non-reducing condition. **Fig. S2:** Uncropped full-length gels and blot results of fibronectin under reducing condition. **Fig. S3:** Uncropped full-length gels and blot results of COL1 under reducing condition. **Fig. S4:** Uncropped full-length gels and blot results of COL3A1 under reducing condition.

## Data Availability

Data supporting the findings of this study are available within the article and its Additional file. The detailed data that support the findings of this study are available on request from the corresponding author [YJ, RST].

## Abbreviations

ADSC: Adipose-derived stem/stromal cell
BMSC: Bone marrow-derived stem/stromal cell
CDPC: Cartilage-derived progenitor cell
COL1, 2, 3: Type I, II, III collagen
DMEM: Dulbecco’s modified Eagle’s medium
ECM: Extracellular matrix
dECM: Decellularized extracellular matrix
FBS: Fetal bovine serum
FGF: Fibroblast growth factor
FN: Fibronectin
GDF: Growth differentiation factor
ITS: Insulin–transferrin–selenium
TGF: Transforming growth factor
