# Supplementary material for: Bioactivity of human adult stem cells and functional relevance of stem cell-derived extracellular matrix in chondrogenesis
Source: Stem Cell Res Ther. 2023 Jun 14;14:160. doi: 10.1186/s13287-023-03392-7 (PMC10268391; doi:10.1186/s13287-023-03392-7)
Supplement: Supplementary file 1 — Additional file 1. Supplementary Materials and Result. Table S1: Primer sequences for real-time RT-PCR. Fig. S1: Western blot results of Fibronectin under non-reducing condition. Fig. S2: Uncropped full-length gels and blot results of fibronectin under reducing condition. Fig. S3: Uncropped full-length gels and blot results of COL1 under reducing condition. Fig. S4: Uncropped full-length gels and blot results of COL3A1 under reducing condition. [file 13287_2023_3392_MOESM1_ESM.docx]

**Supplementary Materials**

**Table S1.** Primer sequences for real-time RT-PCR

| Genes | NCBI  Gene ID | Primer sequences (5’-3’) | | Product size (bp) |
| --- | --- | --- | --- | --- |
| _______________ | ______________ | _____________________________________________________________________ | | __________ |
| COL1A1 | 1277 | Forward | CGATGGATTCCAGTTCGAGTAT | 248 |
|  |  | Reverse | CATCGACAGTGACGCTGTAGG |  |
| COL2A1 | 1280 | Forward | GGATGGCTGCACGAAACATACCGG | 157 |
|  |  | Reverse | CAAGAAGCAGACCGGCCCTATG |  |
| SOX9 | 6662 | Forward | AGCGAACGCACATCAAGAC | 84 |
|  |  | Reverse | CTGTAGGCGATCTGTTGGGG |  |
| AGN | 176 | Forward | AGTCACACCTGAGCAGCATC | 147 |
|  |  | Reverse | AGTTCTCAAATTGCATGGGGTGTC |  |
| RPL13a | 23521 | Forward | CATAGGAAGCTGGGAGCAAG | 157 |
|  |  | Reverse | GCCCTCCAATCAGTCTTCTG |  |
| RNA18S5  (18S) | 100008588 | Forward | GTAACCCGTTGAACCCCATT | 151 |
|  |  | Reverse | CCATCCAATCGGTAGTAGCG |  |
| GAPDH | 2597 | Forward | TGACGCTGGGGCTGGCATTG | 143 |
|  |  | Reverse | GGCTGGTGGTCCAGGGGTCT |  |
| CD44 | 960 | Forward | CTGCCGCTTTGCAGGTGTA | 109 |
|  |  | Reverse | CATTGTGGGCAAGGTGCTATT |  |


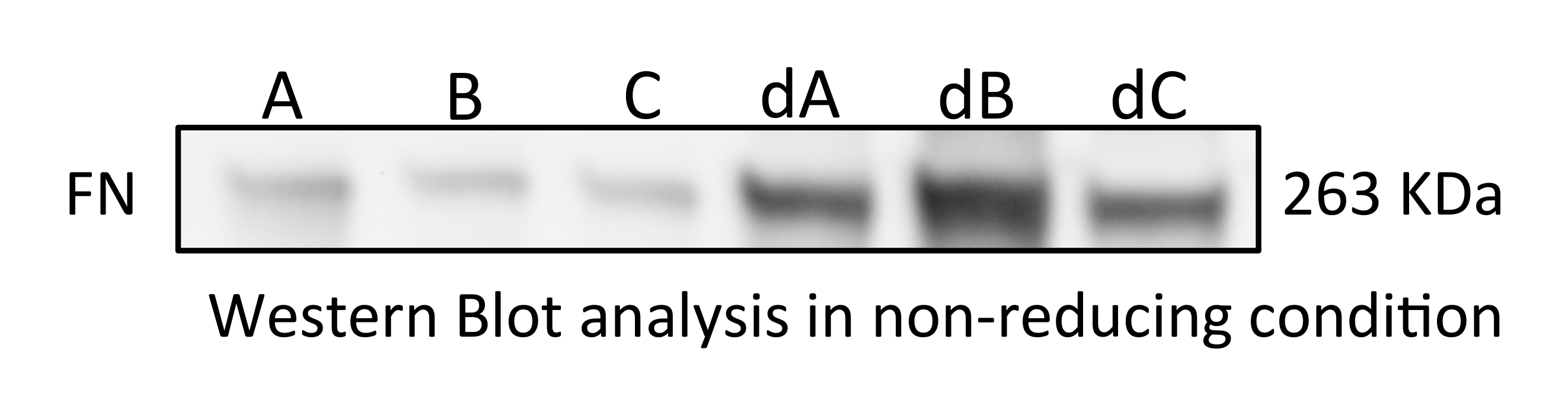


**sFigure 1 Western blot results of Fibronectin under non-reducing condition**

The same amounts of protein (5 μg protein/lane) were loaded into each well. **A**, ADSC cell sheet; **dA**, dECM from ADSC cell sheet; **B**, BMSC cell sheet; **dB**, dECM from BMSC cell sheet; **C**, CDPC cell sheet; **dC**, dECM from CDPC cell sheet. **FN**: fibronectin

**sFigure 2 Uncropped full-length gels and blot results of fibronectin under reducing condition (Figure 4, FN group)**

The same amounts of protein (5 μg protein/lane) were loaded into each well. **A**, ADSC cell sheet; **dA**, dECM from ADSC cell sheet; **B**, BMSC cell sheet; **dB**, dECM from BMSC cell sheet; **C**, CDPC cell sheet; **dC**, dECM from CDPC cell sheet. **FN**: fibronectin; antibody: Abcam, ab281575

**sFigure 3 Uncropped full-length gels and blot results of COL1 under reducing condition (Figure 4, COL1)**

The same amounts of protein (5 μg protein/lane) were loaded into each well. **A**, ADSC cell sheet; **dA**, dECM from ADSC cell sheet; **B**, BMSC cell sheet; **dB**, dECM from BMSC cell sheet; **C**, CDPC cell sheet; **dC**, dECM from CDPC cell sheet. **COL1**: Collagen Type I, antibody: Abcam, ab34710

**sFigure 4 Uncropped full-length gels and blot results of COL3A1 under reducing condition (Figure4, COL3)**

The same amounts of protein (5 μg protein/lane) were loaded into each well. **A**, ADSC cell sheet; **dA**, dECM from ADSC cell sheet; **B**, BMSC cell sheet; **dB**, dECM from BMSC cell sheet; **C**, CDPC cell sheet; **dC**, dECM from CDPC cell sheet. **COL3**: Collagen Type III; antibody: Abcam, ab184993
